# Analysis of the risk transmission mechanism of monetary policy in the monetary and financial service sectors from the perspective of asset-liability management

**DOI:** 10.1371/journal.pone.0292007

**Published:** 2023-10-17

**Authors:** Xuanling Ma, Meng Ji

**Affiliations:** 1 Postdoctoral Workstation, Harbin Bank, Harbin, Heilongjiang Province, China; 2 College of Economics and Management, Harbin Institute of Technology, Harbin, Heilongjiang Province, China; 3 Department of Strategic Development, Harbin Bank, Harbin, Heilongjiang Province, China; University of Baltistan, PAKISTAN

## Abstract

The Silicon Valley Bank crisis that occurred in March 2023 highlights the importance of studying the risk transmission mechanism underlying monetary policy. To explore this mechanism in the monetary and financial services sector from the perspective of asset-liability management, the relationships between asset- and liability-side risks and the corresponding action direction of those risks were empirically studied based on the risk-taking channel theory of monetary policy using publicly available information on 176 Chinese monetary and financial services companies covering the period of 2000 to 2022. The analysis shows that asset-side risks are transmitted through monetary policy as liability-side risks in the monetary and financial service sectors. If the liability side passively takes on these risks, the action direction of monetary policy to asset-side risks is determined; when the liability side actively takes on these risks, monetary policy exerts opposite effects on both types of risks. This conclusion somewhat deepens our understanding of the risk transmission mechanism of monetary policy, which is helpful in better formulating and implementing monetary policy.

## Introduction

Since 2023, monetary and financial services companies (including but not limited to banks, credit cooperatives, loan companies, finance companies, financial leasing companies, and auto financing companies) across Europe and America have shown certain signs of financial crises, which have primarily been attributed to the failure of asset–liability management (i.e., achieving profit maximization through implementing dynamic and optimal management regarding both total quantity and structure, which is generally achieved by selecting and controlling the variety, quantity, maturity, and risk characteristics of assets and liabilities both on and off the balance sheet) to undergo timely adjustments in accordance with changes in monetary policy. The following exemplifies this point: On March 10, 2023, the Silicon Valley Bank, which ranked 16^th^ in assets in the US banking sector, was taken over by the Federal Deposit Insurance Corporation (FDIC) due to insufficient liquidity. Another recent example is Credit Suisse, which ranked 23^rd^ among the global systemically important banks and was acquired by UBS Aktiengesellscharf on March 19, 2023.

To control inflation rates, interest rates have been continuously elevated as a result of the current quantitative easing of monetary policy implemented by the government, and the corresponding abnormal inversion status, in which the short-term interest rate was abnormally higher than the long-term interest rate, has led to a significant increase in financing costs for those monetary and financial services companies. However, several such companies have failed to adjust the total quantity and structure of their assets and liabilities following the changes in monetary policy, resulting in losses from distressed investments [1]. Therefore, further study on the risk transmission mechanism of monetary policy is needed from the perspective of the asset–liability management of monetary and financial services companies.

Previous studies conducted from the perspective of macrosupervision that were focused on the risk transmission mechanism of monetary policy in monetary and financial services have tested several risk transmission mechanisms of monetary policy on the asset side, specifically accounting for the factors of loans, investments, risky assets, impairment reserves, credit capital budgets, and other assets in the monetary and financial services sector [2, 3]. Previous studies also used vector autoregression, income gap, quasinatural experimentation, Bayesian estimation frameworks, nonnested hypothesis tests, Granger causality, and other models to develop and test hypotheses regarding the risk transmission mechanism of monetary policy. These studies have shown that monetary policy was found to transmit risks through interest rate management, portfolio reconfiguration, leverage adjustment, credit decision-making, management incentives for risk transfer, and U.S. dollar financing. Previous studies on risk transmission mechanisms also showed that some monetary policies transmitted risks by expanding assets and deposits or stimulating aggregate demands through wealth channels. Another interesting perspective that has been explored by previous studies is the response of monetary and financial services companies to contrasts between monetary policy and regulatory expectations, and that the specific risk transmission is dependent on the heterogeneity across assets and liabilities, market concentration, enterprise size, liquidity, and leverage ratio [4–14].

Macro- and regulatory-level studies focus only on the asset-side risk of the macro, monetary and financial services sectors as a whole without distinguishing asset-side risks from liability-side risks at the micro and enterprise levels, and therefore these studies inherently suffer from two problems. First, from the perspective of the risk transmission mechanism of monetary policy, assets and liabilities play different roles in the transmission process. From the perspective of the micro and enterprise levels, monetary policy typically acts on liabilities first and then transfers such action to assets. Considering the fact that asset-side risks and liability-side risks are not completely independent of each other, and given changes in monetary policy, there might be a risk transmission mechanism between the asset and the liability sides [15]. Additionally, from the perspective of the economic consequences of risks, the asset and the liability sides inevitably exhibit different characteristics. Asset-side risks are already reflected in the asset portfolio, whereas liability-side risks are directly reflected in the proportion of nondeposit liabilities. Additionally, the high proportion of short-term interbank liabilities at the liability side is the main contributor to monetary and financial services companies crises [16, 17].

Some previous studies begin from the micro and enterprise level perspective, exploring the risk transmission mechanism of monetary policy through loans, investments, and the financing behaviors of specific monetary and financial services companies using various models, including panel data regression, the dynamic two-step system generalized moment method, difference-in-difference (DID), the general equilibrium of open economy, real option, and ownership structure. It was found that monetary policy transferred risks through short-term interest rates, target customers, market power, regulatory means, impairment reserves, and liquidity. Additionally, the different characteristics (e.g., market microstructure, interest rate policy, and enterprise characteristics) of each monetary and financial services company leads to different transmission results: those companies that had major clients and operated in lowly innovative industries with a large size, high market power, sufficient capital, strong liquidity, irreversible investments and inflexible operation were, in general, barely influenced by changes in monetary policy [18–28].

Currently, these micro- and enterprise-level studies still suffer from limitations. First, rather than merely focusing on asset-side risks, these studies have expanded the analysis to include the source of financing perspective, such as bond market financing [19], retained earnings [21], and ownership differences [28], but they have not examined the risk transmission of monetary policy from the perspective of each individual item within the liability side. Second, previous studies have mainly used time series data [18–21, 24–27], focusing on obtaining a general conclusion for all companies in the monetary and financial services sector as a whole, but few empirical studies have conducted a more detailed analysis at the company-specific level. Additionally, micro- and enterprise-level studies are generally more detailed than macro- and regulatory-level studies. The risk transmission mechanism of monetary policy is relatively fuzzy, and no extant studies have been performed from the combined perspective of both the macro- and regulatory-level and the micro- and enterprise-level.

There is a growing body of theoretical research regarding the risk-taking channel of monetary policy in the monetary and financial services sectors [29], but little literature has distinguished between the asset- and liability-side risk-taking of monetary and financial services enterprises. The research gap relates to how monetary policy acts on the asset- and liability-side risk-taking of monetary and financial services enterprises. The traditional theory regarding monetary policy transmission mechanisms assumes that monetary and financial services enterprises are risk-neutral and ignores the impact of the monetary financial services sector’s own risk perception and risk tolerance in the transmission effect of monetary policy [30]. The fact that the policy rate affects the risk perception or risk tolerance of the monetary and financial services sector creates a risk-taking channel for monetary policy, which in turn affects the riskiness of asset portfolios and asset pricing, the cost of funds, and the financing conditions [31]. The risk-taking theory of monetary policy is adopted to analyze the impact of policy interest rates on risk-taking in the monetary financial service sectors under two different scenarios of endogenous and exogenous leverage, as well as on the equilibrium results under different market structures [32]. The interaction assessment of macroeconomic variables such as policy rate and risk-taking in the vector autoregression method at the macro level [33] and the study of the relationship between monetary policy and risk indicators such as nonperforming assets using the dynamic panel data model generalized method of moments and other micro-level methods [34] have not provided clarity on how monetary policy affects risk-taking on either the asset or the liability side and have failed to determine risk-taking. The main reason for this is that both asset- and liability-side risk-taking are endogenous and need to be examined in a unified framework to explore the changes in the two under external shocks, and there has been little work conducted to explore this issue under the framework of the monetary policy transmission mechanism. In view of this, the risk-taking channel theory of monetary policy is adopted to study this blind spot and uncover the corresponding transmission mechanism. Exploring and answering the above questions not only helps to deepen the theoretical understanding of monetary policy risk-taking channels but also assists in improving the formulation and implementation of monetary policy in a more targeted manner.

In this study, using monetary and financial services companies in China as the research sample, the following two questions are explored: (1) Does monetary policy act upon the liability-side risks of monetary and financial services before transmitting to asset-side risks? (2) Does monetary policy act upon the asset-side risks in the same direction as it does upon the liability-side risks of monetary and financial services? This study contributes to the literature in the following ways. First, the risks faced by monetary and financial services companies are clearly divided into asset-side risks and liability-side risks, and the transmission mechanisms of monetary policy to each of these risk dimensions are analyzed. Additionally, an empirical analysis is performed using panel data from the monetary and financial services company level rather than using time series data to further examine the risk transmission mechanism of monetary policy at a more detailed, micro and enterprise level. Finally, the risk transmission relationships between the liability- and asset-side in the risk transmission mechanism of monetary policy in the monetary and financial services sector are determined on the basis of the risk-taking channel theory of monetary policy, thus actively combining perspectives on both the macro and regulatory levels and the micro and enterprise levels.

The remainder of the study is organized as follows: In Section II, the theoretical hypotheses on the risk transmission mechanism of monetary policy in the monetary and financial services industry are proposed by reviewing and analyzing the previous performed studies and literature. In Section III, the corresponding testing models are constructed and the relevant variables and data used are described. In Section IV, the empirical results of relevant models analyzed, and the results for those hypotheses are tested in Section II. Given the hypothesis and test results, Section V conducts a further discussion and analysis from the perspective of asset–liability management. Section VI presents the conclusions, implications, and future developments and expectations.

## Literature review and hypothesis development

### Risk-taking channel theory of monetary policy

The risk-taking channel theory of monetary policy, in which risk-taking is defined as the extent to which monetary and financial services enterprises monitor their lending was proposed in 2010. In this theory, monetary policy affects risk-taking through two pathways that work in opposite directions: the interest rate transmission effect and the risk transfer effect [35]. The theory is established on the basis of two assumptions. First, financial institutions have limited liability and choose the amount of effort they expend in loan supervision, which can then serve as a measure of risk-taking. Second, the cost of liabilities is affected by risk-free interest rates for the monetary and financial service sectors. Based on these two assumptions, monetary policy affects the risk-taking of financial institutions via two effects: the interest rate transmission effect and the risk transfer effect. The interest rate transmission effect suggests that the reduction in the underlying interest rate benchmark leads to a reduction in the loan interest rate, which reduces the total income of a given asset portfolio and therefore leads to a reduced effort in supervising loans and improving the level of risk-taking. The risk transfer effect suggests, all things being equal, that a reduction in the underlying interest rate benchmark reduces the cost of liability, and if a loan can be recovered, profit increases. Therefore, monetary and financial services companies have more incentive to supervise the loan, which reduces the level of risk. The interest rate transmission effect and risk transfer effect both lead to an adverse relationship between monetary policy and the risk-taking of monetary and financial services companies.

Risk-taking channel theory can only be used to analyze asset-side risks and do not take into account liability-side risks or the interactions between asset-side risks and liability-side risks. However, it has been observed that monetary and financial services companies respond to changes in monetary policy by adjusting both their asset and their liability structures. Based on this fact, both the asset- and the liability-side of monetary and financial services companies are responsive to the impact of monetary policy, and a transmission relationship may exist between them. Risk-taking on the liability side is divided into active risk-taking and passive risk-taking. Active risk-taking means that when monetary policy changes, monetary and financial services companies actively adjust their risk level to maximize profits, while passive risk-taking means that in case of changes in monetary policy, the monetary and financial services companies do not take the initiative to respond. However, the liability-side risks change spontaneously because the liability side is dominated by deposits from individual and corporate savings, which are subject to fluctuations. When monetary policy is loose/tight, deposits, as a fundamental part of the money supply, increase/decrease accordingly, and as the proportion of deposits increases/decreases, the liability-side risks decrease/increase accordingly.

### Liability-side passive risk-taking

It has been concluded that when monetary policy is stringent, the underlying interest rate benchmark drops, leading to a decrease in the money supply, a decrease in the liability-side deposits of monetary and financial services companies, and an increase in the level of reliance on interbank liability financing for those companies; in contrast, when monetary policy is lax, the underlying interest rate benchmark rises, leading to an increase in the money supply, an increase in the liability-side deposits of monetary and financial services companies, and ultimately causing a decreased reliance on interbank liability financing for those companies [36]. Therefore, this study begins with an analysis of the relationship between monetary policy and asset-side risks, and Hypothesis 1 is proposed as follows:

Hypothesis 1: Considering that the liability side of monetary and financial services companies inevitably bears passive risks, the flexibility of monetary policy has an adverse effect on asset-side risks; in other words, a laxer monetary policy reduces asset-side risks, whereas a more stringent monetary policy increases asset-side risks.

### Active risk-taking on the liability side

Two contradictory effects were observed for the risk transmission mechanism between the liability-side and the asset-side of monetary and financial services companies. One of these effects is the risk transfer effect, which suggests that liability-side risks are directly transmitted to the asset side, and the higher risks and higher costs of the liability side are covered by the higher returns of the asset side. Therefore, asset-side risks are positively correlated with liability-side risks; in other words, the higher the risks generated from the liability side are, the higher the risks transferred to the asset side. The other effect is the risk dispersion effect. The risk dispersion effect suggests that when the liability-side risks rise, the monetary and financial services companies tend to withhold any increase in their exposure to risky assets (such as risky investments and risky loans) within their investment portfolio and rather tend to pursue a more stable return in exchange for the higher expected returns associated with increased exposure to risky assets; in other words, the higher that the risks generated from the liability side are, the less risk that is taken on the asset side. However, regardless of the recoverability of loans to external customers, interbank liability financing should always be fully repaid. This stringent, full-payment nature of interbank financing on the liability side, in turn, reinforces the risk dispersion effect and would outweighs the risk transfer effect. Thus, it has been observed that the higher that the risks generated from the liability side are, the lower the risks taken on the asset side [37]. In this study, the further assessment of the relationship between the proportion of nondeposit liabilities of monetary and financial services companies and asset-side risks is called for, and Hypothesis 2 is proposed as follows:

Hypothesis 2: The higher that the proportion of nondeposit liabilities of monetary and financial services companies is, the higher the degree of effort required for loan supervision. That is, the risks on the liability side are transmitted to the risks on the asset side, but risks on the liability side have an adverse effect on the risks on the asset side.

When monetary policy is lax, the money supply increases, and the deposits on the liability side of monetary and financial services companies increase. As a statutory requirement within the monetary and financial service sectors, deposits obtained from individuals and enterprises are protected by statutory insurance policies. When the recoverability of distressed investments or loans suffers from unexpected turbulences in the general economy, the losses are reduced by the statutory insurance policies, and the net losses suffered by monetary and financial services companies are mitigated accordingly [38]; therefore, the liability-side risks are reduced by statutory insurance [39]. Based on the above discussion, Hypothesis 3 is proposed as follows:

Hypothesis 3: With an increase in the underlying interest rate benchmark, the nondeposit liabilities born by monetary and financial services companies increase accordingly. That is, a stringent monetary policy increases liability-side risks, whereas a lax monetary policy reduces liability- side risks.

According to the risk-taking channel theory of monetary policy, the impact of monetary policy on the asset-side risks of monetary and financial services companies depends on the relative weights of the interest rate transmission effect and the risk transfer effect [40]. In this study, the risk transfer effect as tested in Hypothesis 2 is combined with the risk dispersion effect as tested in Hypothesis 3. In a quantum easing of monetary policy, the deposits on the liability-side increase and the sources of liabilities become more stable, which encourages monetary and financial services companies to actively seek higher returns from the asset-side by taking increased risks. The interest rate transmission effect and risk dispersion effect outweigh the risk transfer effect. Thus, a lax monetary policy leads to increase the asset-side risks, whereas a stringent monetary policy leads to a decrease in asset-side risks. Therefore, Hypothesis 4 is proposed as follows:

Hypothesis 4: With an increase in the underlying interest rate benchmark, the efforts required for loan supervision by monetary and financial services companies increase. That is, a stringent monetary policy decreases asset-side risks, whereas a lax monetary policy increases asset-side risks.

## Materials and methods

### Modeling

Referring to past practice [28, 41], the following models were used in this study:

$$ARISK_{i,t}=\alpha_{0}+\alpha_{1}MP_{t}+\rho C_{i,t}+\delta_{i}+\theta_{t}+\varepsilon_{i,t},$$ (1)

$$LRISK_{i,t}=\beta_{0}+\beta_{1}MP_{t}+\sigma C_{i,t}+\delta_{i}+\theta_{t}+\zeta_{i,t},$$ (2)

$$ARISK_{i,t}=\gamma_{0}+\gamma_{1}LRISK_{i,t}+\gamma_{2}MP_{t}+\tau C_{i,t}+\delta_{i}+\theta_{t}+\vartheta_{i,t},$$ (3)

where *ARISK*_*i*,*t*_ indicates the asset-side risks; *LRISK*_*i*,*t*_ denotes the liability-side risks; *MP*_*t*_ represents the monetary policy; *C*_*i*,*t*_ is a control variable; *δ*_*i*_ is the individual heterogeneous feature; *θ*_*t*_ is the time-fixed effect; and *ε*_*i*,*t*_, *ζ*_*i*,*t*_, and *ϑ*_*i*,*t*_ are error terms.

Model (1) was designed to test whether monetary policy had a significant impact on the asset-side risks of monetary and financial services companies. Model (2) was designed to test whether the liability-side risks born by monetary and financial services companies are significantly affected by monetary policy, and Model (3) was used to test whether the liability-side risks born by monetary and financial services companies significantly influence the asset-side risks. Taking Models (1) through (3) as a whole, whether risks were transmitted to the asset side through the liability side for monetary and financial services companies.

### Variables

#### Monetary policy

Referring to past practice [42], whether the underlying deposit interest rate benchmark (DRATE) and interbank pledged a 7-day reverse repo rate (R007) was used as the measure of monetary policy. In this empirical study, DRATE takes the underlying deposit interest rate benchmark stipulated by the People’s Bank of China, while R007 is the short-term pledged reverse repo rate in the China interbank market used to capture the changes in monetary policy from the perspective of short-term fluctuations.

At the same time, unlike central banks in developed nations, the People’s Bank of China (i.e., China’s central bank) operates a reserve system, where not all deposits can be used for loan issuance, and a portion of the deposits are in the form of legal reserve deposits, which are made by monetary and financial service enterprises to the central bank. In this study, the legal reserve ratio (RESERVE) was adopted as a proxy variable for monetary policy.

#### Risk-taking

Referring to practice [43], the proportion of risk-weighted assets to total assets was selected to represent the asset-side risks. The level of risks taken by a monetary and financial service company is directly reflected by the portion of risky assets within its investment portfolio; thus, the proportion of risk-weighted assets could also indicate a company’s level of active risk-taking. In addition, loan loss provisions (“LLP”) and nonperforming loans (“NPL”) belong to the passive risk-taking of the monetary and financial services sector. Thus, the LLP or NPL ratio is an ex post measure representing the risk level faced by monetary and financial services enterprises that does not adequately reflect changes in the risk appetite and risk behavior of monetary and financial services enterprises and does not directly characterize the asset-side risk-taking of the monetary and financial services sector.

Deposits are generally guaranteed by a deposit insurance system, but interbank liability financing is not insured. Interbank liabilities include the interbank borrowing and selling of repurchased financial assets, which are riskier than deposits. Although China’s deposit insurance regulations, which have been in effect since May 1, 2015, provide for the full repayment of all insured deposit accounts of the same depositor at the same insured institution covering the combined amount of principal and interest of the funds within 500,000 CNY, it is argued in this study that the impact of the deposit insurance factor is extremely small. First, from a practical point of view, China had an implicit deposit insurance system in place prior to the establishment of the official system because bank deposits have been covered by the implicit guarantee of national credit since before the establishment of the deposit insurance system in China. Second, following the formal establishment of the deposit insurance system, bank deposits could be covered in full by the vast majority of depositors, which is basically equivalent to full deposit insurance. Considering the facts that deposits are unlikely to be run at the first sign of a crisis and that runs on interbank liabilities have played a key role in the bankruptcy and failure of many financial institutions, many recent studies have adopted and utilized this method of measuring liability-side risk-taking, given that monetary and financial services enterprises with a high proportion of nondeposit liabilities are more susceptible to changes in the level of market liquidity, which makes them riskier. With reference to practice [44], this study also uses the ratio of nondeposit liabilities to total assets as a measure of liability-side risks.

#### Control variables

Referring to past practice [45], the following four control variables were chosen: enterprise size (SIZE), return on assets (ROA), operational efficiency (OPERA), and leverage ratio (LEVER). A dummy variable, GDP, was introduced to control the macrolevel influencing factors. This variable is designed to reflect the GDP growth rate, and it takes the value of 1 if the GDP growth rate of the current year is greater than that of previous year and 0 otherwise.

In addition, to incorporate qualitative factors such as risk culture and corporate governance, a dummy risk management variable, COMMITTEE, was introduced to control for microlevel factors. If the risk management committee is headed by an independent director, more than half of its members are nonexecutive directors, and it meets more than four times a year, then it is assigned a value of 1 and a value of 0 otherwise.

## Results

### Descriptive statistics

With 176 monetary and financial services companies in China between the years between 2000 and 2022 composing the study sample, all monetary and financial services company variables were subjected to 1% quantile and 99% quantile standardization to mitigate the effect of extreme values. The sample in this study is drawn from the Wind Database (https://www.wind.com.cn/). The banks operating in China from 2000 to 2022 were selected from this database, and those with observations of less than 4 years old were excluded to obtain the final sample of 176 banks. Table 1 displays the descriptive statistics of the variables mentioned above.

**Table 1:** Descriptive statistics for variables.

Variables	Description	Mean	Standard deviation	Minimum	Maximum
**DRATE**	underlying interest rate benchmark for a one-year deposit	2.693	0.982	1.782	7.248
**R007**	Interbank pledged 7-day reverse repo rate	3.13	0.733	1.528	4.712
**RESERVE**	CNY legal reserve contribution ratio	12.283	4.481	6	18.5
**ARISK**	Risk-weighted assets as a percentage of total assets	60.331	13.326	23.167	102.868
**LRISK**	Nondeposit liabilities as a percentage of total liabilities	22.102	11.941	2.285	64.594
**SIZE**	The logarithmic value of total assets	7.814	2.173	3.309	12.274
**ROA**	Return on assets, in %	1.147	0.817	-0.364	2.655
**OPERA**	Operation efficiency, as calculated by operating costs divided by total revenue	37.723	10.014	18.165	116.128
**LEVER**	Leverage ratio, as calculated by shareholder’s equity divided by total assets	7.346	2.546	2.523	42.063
**GDP**	Dummy variable reflecting whether the GDP growth rate of the current year is greater than that of the previous year	0.44	0.805	0	1
**COMMITTEE**	Dummy variable reflecting whether the risk management committee is headed by an independent director, more than half of its members are nonexecutive directors, and it meets more than four times a year	0.37	0.746	0	1

Source: Author’s calculations.

### Empirical results of monetary policy risk transmission

In this study, the sample was further divided into three distinct time periods to reflect two significant changes in monetary policy. Period I represents the years between 2000 and 2007, Period II represents the years between 2008 and 2014, and Period III represents years between 2015 and 2022.

Before the global financial crisis of 2008, the liabilities of monetary and financial services companies in China derived mainly from deposits, and these companies tended not to take active risks. Since the global financial crisis of 2008, Chinese monetary and financial services companies have begun actively take some risks, and the risk transmission mechanism of monetary policy has become increasingly prominent. Beginning in 2015, with the change in the money supply mode, the liability-side risks became significantly higher than those of previous periods, and the proportion of nondeposit liabilities increased rapidly. Thus, in this study, the monetary policy (MP) variable takes DRATE for the first two of the three periods and R007 for the last period to better simulate the transmission effect.

Table 2 shows the regression results for the 176 monetary and financial services companies in China for the years of 2000 to 2022.

**Table 2:** Regression results.

Periods Results Variables	2000–2007			2008–2014			2015–2022		
	(1)	(2)	(3)	(4)	(5)	(6)	(7)	(8)	(9)
	ARISK	LRISK	ARISK	ARISK	LRISK	ARISK	ARISK	LRISK	ARISK
**MP**	14.711*** (3.529)	16.361*** (1.292)	15.167*** (3.817)	-6.571*** (2.263)	14.259*** (1.319)	-5.184*** (2.146)	-7.466*** (0.724)	3.967*** (0.491)	-7.347*** (0.648)
**LRISK**			-0.207** (0.102)			-0.172*** (0.031)			-0.326*** (0.046)
**SIZE**	20.144*** (4.122)	22.047*** (1.206)	20.710*** (3.771)	-3.258*** (1.101)	17.093*** (0.731)	-1.967* (1.122)	0.514 (0.973)	11.602*** (0.715)	1.712* (0.983)
**ROA**	-9.146*** (1.938)	-7.786*** (0.553)	-9.961*** (1.649)	6.189*** (0.874)	-0.975* (0.545)	5.282*** (0.946)	-0.132 (0.614)	-3.752*** (0.639)	-0.821 (0.627)
**OPERA**	-0.201*** (0.063)	0.126*** (0.017)	-0.371*** (0.054)	0.432*** (0.053)	-0.129** (0.043)	0.271*** (0.025)	-0.014 (0.029)	-0.046** (0.028)	-0.017 (0.029)
**LEVER**	3.061*** (0.491)	0.355*** (0.092)	2.916*** (0.518)	0.961*** (0.078)	-0.804*** (0.086)	0.776*** (0.097)	2.864*** (0.156)	-1.162*** (0.162)	2.439*** (0.178)
**GDP**	-9.944 (9.733)	-8.769* (4.724)	-10.684 (9.322)	2.302*** (0.508)	-0.221 (0.469)	2.338*** (0.319)	-0.147 (0.383)	0.115 (0.281)	-0.366 (0.327)
**COMMITTEE**	-11.569 (9.573)	-6,257 (5.612)	-11.702 (6.631)	2.746*** (0.355)	-1.621 (0.312)	2.393*** (0.383)	-0.618 (0.289)	0.091 (0.184)	-0.322 (0.168)
**Constant term**	-231.263*** (57.307)	-283.501*** (19.476)	-232.161*** (56.216)	92.613*** (18.395)	-236.639*** (13.458)	70.318*** (18.129)	52.399*** (13.109)	-117.409*** (9.352)	39.441*** (13.827)
**Entity-fixed effects**	Yes	Yes	Yes	Yes	Yes	Yes	Yes	Yes	Yes
**Time-fixed effects**	Yes	Yes	Yes	Yes	Yes	Yes	Yes	Yes	Yes
**Observations**	112	112	112	506	506	506	1160	1160	1160

Note: Standard errors are displayed in brackets; and

*, **, *** represent significant at the levels of 10%, 5% and 1%, respectively.

Source: Author’s calculations.

## Discussion

In general, based on the regression analysis results, all four hypotheses proposed in this study were supported by empirical data through the use of statistical methods, which supports that claim that monetary policy first interacts with the liability-side risks of monetary and financial services companies and then transmits to the asset-side risks. The results are consistent with the conclusions [36]. At the same time, the result also show that if monetary policy leads to increased liability-side risks for monetary and financial services companies, it also leads to decreased asset-side risks for those companies, and vice versa. These findings are similar to those observed in [37, 33]. A further breakdown analysis for the three distinctive time periods is listed as follows.

For Period I, which used data between 2000 and 2007, the regression results are presented in Columns (1) through (3) of the Table 2 regression results. It is quite clear that the variable MP is significant at the 99% confidence level for all three regression models. Thus, based on the hypothesis, monetary policy indeed exerted a significant influence on the asset- and liability-side risks of monetary and financial services companies between 2000 and 2007. Additionally, the regression coefficient of MP was positive in all three regression models, indicating that the asset- and liability-side risks interact in the same direction as the changes in monetary policy. That is, as monetary policy becomes more lax, asset- and liability-side risks both increase, and vice versa. Past empirical findings have suggested that loose monetary policy rates increase bank risk-taking [30], but the current findings presented in this study indicate that further asset- and liability-side risks both increase as well.

From the perspective of the liability side, which mainly consists of deposits, when the monetary policy is stringent, deposits are expected to decrease, and the proportion of nondeposit liabilities passively increases with other factors being equal. In contrast, when the monetary policy is lax, deposits are expected to increase, and nondeposit liabilities passively decrease with other factors being equal. On the other hand, from the perspective of the asset side, the decrease in the underlying deposit interest rate benchmark reduces the liability cost of monetary and financial services companies. If their distressed investments and loans could be even partially recovered, their profits would increase, creating a motive for them to put effort into monitoring the recoverability of investments and loans, which in turn would reduce the risks associated with these investments as well as the asset-side risks. This result supports Hypothesis 1. At the same time, it can be observed that the liability side plays an intermediate role in the transmission mechanism of monetary policy to the asset side in an adverse relationship: the greater the level of risk taken on the liability side is, the lower the level of risk taken on the asset side.

For Period II, which used the data between 2008 and 2014, the regression results are presented in Columns (4) through (6) of Table 2. For Period III, which used data covering the period of 2015 to 2022, the regression results are presented in Columns (7) through (9) of Table 2. In contrast to the findings of Period I, the regression results presented in Columns (4) and (7) for Period II show a significantly negative coefficient for the explanatory variable liability-side risks of monetary and financial services companies. This result indicates that the liability-side risks had a significant adverse effect on the asset-side risks, which was consistent with Hypothesis 2.

Additionally, from the results displayed in Columns (5) and (8) of Table 2, monetary policy can be seen to have a significantly positive correlation with the liability-side risks of monetary and financial services companies, suggesting that a more lax monetary policy reduces liability-side risks, while a stringent monetary policy increases liability-side risks; thus, Hypothesis 3 is also supported.

Taking both the MP and LRISK variables into account, the regression results are shown in Columns (6) and (9) in Table 2. The results showed that, when considering both the variables of monetary policy and liability-side risks, asset-side risks interact in contrast to these two variables, as the coefficients of these two variables are both negative and significant. In other words, a more lax monetary policy or a decrease in liability-side risks leads to an increase in asset-side risks, and a more stringent monetary policy or an increase in liability-side risks lead to a decrease in asset-side risks. The regression results also support the validity of Hypothesis 4.

(3) Comparing the regression results displayed in Columns (4), (6), (7) and (9), the coefficients of liability-side risks can be seen to remain negative and significant for asset-side risks, indicating that liability-side risks play an intermediate role in the transmission mechanism of monetary policy to asset-side risks with all other conditions being equal. In other words, monetary policy significantly interacts with the asset- and liability-side risks of monetary and financial services companies, and at the same time, the risk transmission mechanism of monetary policy suggests that monetary policy transmits risks to the asset side by influencing liability-side risks. In addition, a more lax monetary policy decreases the level of liability-side risks, which, in turn, leads to an increase in the level of asset-side risks, and vice versa. Unlike past findings showing that monetary policy transmission becomes weaker with increased market concentration [33], our findings indicate that monetary policy works in different directions for asset-side and liability-side risk-taking rather than uniformly working in one direction. Therefore, the asset- and liability-side should be examined separately to assess the impact of their risk-taking on monetary policy.

When in a lax monetary environment, the deposits of monetary and financial services companies increase. Given that these deposits are subject to fewer fluctuations than other liabilities (such as interbank repurchases) and are protected by statutory deposit insurance, the liability-side risks are reduced. As a source of liability financing that is subject to fewer fluctuations, deposits create a precondition for monetary and financial services companies to be actively exposed to higher levels of risk and thus gain higher returns on the asset side. On the one hand, monetary and financial services companies with high deposits often face an excess of liquidity, which negatively affects their profitability; thus, they are motivated to actively take more risks on the asset side [46]. On the other hand, the loan amount was an important evaluation benchmark for monetary and financial services companies, and the steady increase in deposits also creates incentives for monetary and financial services companies to provide riskier loans [47]. With changes to monetary policy, the liability- and asset-side risks both change as well.

## Conclusions and implications

### Conclusions

Based on the publicly disclosed data of 176 monetary and financial services companies in China over the years of 2000 to 2022, the risk transmission mechanism of monetary policy has been discussed from the perspective of asset-liability management based on the risk-taking channel theory of monetary policy, and the following findings have been drawn:

(1) The influences of monetary policy on the asset- and liability-side risks of monetary and financial services are not independent of each other. The effect of changes in monetary policy is transmitted to asset-side risks through liability-side risks. However, in an adverse relationship between asset- and liability-side risks, an increase in the level of liability-side risks leads to a decrease in the level of asset-side risks.

(2) Given that the liability side of monetary and financial services companies bears passive risks, monetary policy exerts a negative effect on asset-side risks: lax monetary policy decreases asset-side risks, while a stringent monetary policy increases asset-side risks.

(3) Given that the liability side of monetary and financial services also actively takes on risks, monetary policy has a contradictory effect on asset-side risks and liability-side risks; in other words, a more lax monetary policy increases asset-side risks but decreases liability-side risks, while a more stringent monetary policy decreases asset-side risks but increases liability-side risks.

### Managerial implications

Based on the findings above, the following implications are proposed:

(1) For the implementation of monetary policy and related macro supervision activities, standard setting committees and regulatory authorities are encouraged to incorporate new data and new procedures from a regulatory perspective to oversee the compliance and asset- and liability-side risks of monetary and financial services companies. As demonstrated in this study, changes in monetary policy have opposite effects on the asset- and liability-side risks of monetary and financial services companies. Thus, more indicators, such as core liabilities, interbank liabilities, asset allocation and asset quality, are needed to ensure that both asset- and liability-side risks are substantially under control. Especially in the current situation of continuous stringent monetary policy applied in the major developed economies, liability-side risks increase as active risk-taking on the liability side increases (as shown in this study), and regulatory authorities are encouraged to promote the prevention of unnecessary risk exposure given the narrowing interest margin and the pressure to expand profits in the monetary and financial services sectors.

(2) Changes in monetary policy exert their own influences on each monetary and financial services company, and such companies are encouraged to consider these factors when formulating their own risk statements. This study confirms that a risk transmission mechanism of monetary policy exists for monetary and financial services companies, and monetary policy also affects the risk preference of monetary and financial services companies at both the individual company and the sector levels. The study showed that changes in monetary policy and any corresponding changes in underlying deposit interest rate benchmarks follow the transmission mechanism and affect the implementation of risk statements. Monetary and financial services companies should create their own risk statements based on the sector consensus. In these risk statements, relevant activities and procedures are presented to illustrate how the monetary and financial services companies aim to achieve such stated risks. Items in the risk statement should include, but are not limited to, risk preference, risk policies, risk limits, management review processes, internal control procedures, and related information systems to support the implementation of such risk statements. The risk statement should also be considered at the strategic, business decision-making and operational levels to ensure its full implementation throughout the company.

(3) The financial services sector is encouraged to pursue innovative actions regarding the current asset-liability management theories. This study demonstrates that the liability-side risks of monetary and financial services are transmitted to asset-side risks, indicating that the traditional asset-liability management theory, which assumes that assets and liabilities are two independent portfolios, is flawed when accounting for the risk transmission mechanism. Therefore, as stated above, regulatory authorities are encouraged to incorporate new indicators to oversee the unnecessary risk exposures of each individual monetary and financial service company, and individual monetary and financial service companies are encouraged to take innovative actions to develop their own asset-liability management methods. On the one hand, the use of internal and external pricing and analysis tools to provide recommendations on low-cost sources of liabilities and thus reduce liability-side risks is encouraged. On the other hand, asset-side risks should also be carefully considered as the customer base expands, especially in the areas of improving marketing efficiency, enhancing customer stickiness, enriching product functions and driving transactions.

### Limitations and future directions

This study extends the research on the risk transmission mechanism of monetary policy in the monetary and financial services sector and applies the risk transmission mechanism to China’s empirical data for the period of 2000 to 2022, which broadened the previous literature into the emerging market of China and incorporates over 20 years of data regarding Chinese monetary and financial services companies. The research findings have important significance for both theoretical researchers and regulatory authorities and for the risk and asset-liability management of the monetary and financial services industry. However, this study still has its own limitations. For example, the data used in this study were mainly taken from the publicly disclosed information of these monetary and financial services companies, and the sample was not large. Only a few control variables were used to describe the characteristics of monetary and financial services companies, and the standardization of the variables might not capture the actual results.

The following research directions are suggested for further development: (1) Research on the risk transmission mechanism of monetary policy can be further analyzed in terms of each of the main risk factors for both asset- and liability-side risks in an empirical study. In this study, the overall risk transmission mechanism of monetary policy in monetary and financial services companies was investigated. Such research can be furthered by analyzing the main risk factors on the asset- or liability-side, e.g., the portion or absolute amount of interbank loans. (2) Qualitative factors such as risk culture have not been incorporated into the current research on the risk transmission mechanism of the monetary policy of monetary and financial services companies. The incorporation of qualitative factors such as corporate governance is also a future research direction.

## Supporting information

S1 Data (XLSX)
